# Fully Printed Wearable Vital Sensor for Human Pulse Rate Monitoring using Ferroelectric Polymer

**DOI:** 10.1038/s41598-018-22746-3

**Published:** 2018-03-13

**Authors:** Tomohito Sekine, Ryo Sugano, Tomoya Tashiro, Jun Sato, Yasunori Takeda, Hiroyuki Matsui, Daisuke Kumaki, Fabrice Domingues Dos Santos, Atsushi Miyabo, Shizuo Tokito

**Affiliations:** 1 Research Center for Organic Electronics (ROEL), Graduate School of Science and Engineering, Yamagata University, Yonezawa, Yamagata 992-8510 Japan; 2 Arkema-Piezotech, 63493 Pierre-Benite Cedex, France; 3 Arkema K. K., 93, Chudoji, Awatacho, Shimogyo, Kyoto, 600-8815 Japan

## Abstract

The ability to monitor subtle changes in vital and arterial signals using flexible devices attached to the human skin can be valuable for the detection of various health conditions such as cardiovascular disease. Conventional Si device technologies are being utilised in traditional clinical systems; however, its fabrication is not easy owing to the difficulties in adapting to conventional processes. Here, we present the development of a fully printed, wearable, ferroelectric-polymer vital sensor for monitoring the human pulse wave/rate on the skin. This vital sensor is compact, thin, sufficiently flexible, and conforms to the skin while providing high pressure sensitivity, fast response time, superior operational stability, and excellent mechanical fatigue properties. Moreover, the vital sensor is connected to a communication amplifier circuit for monitoring the pulse waves with a wireless sensing system. This sensor system can realise the development of new healthcare devices for wearable sensor applications.

## Introduction

Wearable and flexible electronics, such as ion-sensor arrays, biosensor chips, and vital sensor patches^1–4^, have attracted considerable attention due to their potential applications in healthcare. Vital sensors must be enabled constantly for continuous healthcare monitoring. This can be achieved by fabricating wearable sensors. Among the various types of wearable and flexible electronics available for vital sensors, piezoelectric pressure sensors have been extensively studied^5,6^. Generally, these sensors are used for human motion analysis in applications including pulse wave sensors and motion sensors for motion capture^7–12^. The monitoring and analysis of any information on human physiology is significant in the fields of healthcare and academic science. Moreover, in recent years, printing process technologies have attracted considerable attention as new methods for sensor fabrication. When compared with conventional photolithography and vacuum deposition processes, fabrication processes based on printing technology can drastically improve material utilisation and reduce waste while minimising the processing cost^13–15^.

For the realisation of flexible printed pressure sensors. Poly(vinylidene fluoride) (PVDF) and its copolymer, poly(vinylidene fluoride-co-trifluoroethylene) [P(VDF-TrFE)], are some of the most common ferroelectric materials used in piezoelectric sensors^9,16–20^. For example, ferroelectric/piezoelectric materials are used in ultrasonic transducers^21,22^, microelectromechanical devices, and actuators^23–25^, as well as in pressure and strain sensors. Owing to their ferroelectricity, these polymers have high dielectric permittivity and significant remnant polarization^26–28^. In particular, the P(VDF-TrFE) copolymer is advantageous in printing processes because it is soluble in polar solvents and can adapt to the solution/printing processes. A sensor fabricated using this copolymer can monitor various vital signs unerringly for 24 h because of the mechanical and chemical stabilities of the copolymer^22,29–31^. Printed, vital P(VDF-TrFE) sensors can be utilised in healthcare applications because the acoustic impedance of P(VDF-TrFE) is considerably close to that of a living organism. However, heretofore, there have been no reports on the monitoring of signals using wearable printed vital sensors. In addition, the wireless monitoring of vital signals has not been studied. For now, PVDF homopolymer precursor has been used to monitor vital signs^32–38^. On the other hand, very few studies have reported the detection of vital signals with sensor devices based on printing technologies. Furthermore, recent works on the effects of polar solvents used for the insulating layers in spin casting have demonstrated that a large dipole moment enhances the ferroelectric characteristics; however, there are few studies on the ferroelectricity of the printed copolymer layers for monitoring the human vital signs^39^. In addition, the detailed effects of the dipole moments of the polar solvents on the printing process have not been reported.

In this paper, we present a fully printed, wearable vital sensor made of ferroelectric polymer for monitoring the human pulse wave/rate on the skin. This sensor is compact (~4 mm^2^), thin (~3 μm), and sufficiently flexible, and conforms to the skin while providing high pressure sensitivity (~0.025 MPa), fast response time (~0.2 s), superior operational stability, and excellent mechanical fatigue properties. The surface morphology of the printed P(VDF-TrFE) layer, as a pressure-detecting layer, was improved by using an optimum polar solvent; the optimisation realised satisfactory ferroelectricity, which was approximately 7.0 μC cm^−2^ of polarization, and high pressure sensitivity. The pressure sensor was connected to a specifically designed wireless sensing system for wireless monitoring of the pulse wave/rate. This sensor system can realise the development of novel healthcare devices for continuous health/wellness monitoring applications.

## Results

### Device design and fabrication

**Figure 1:**
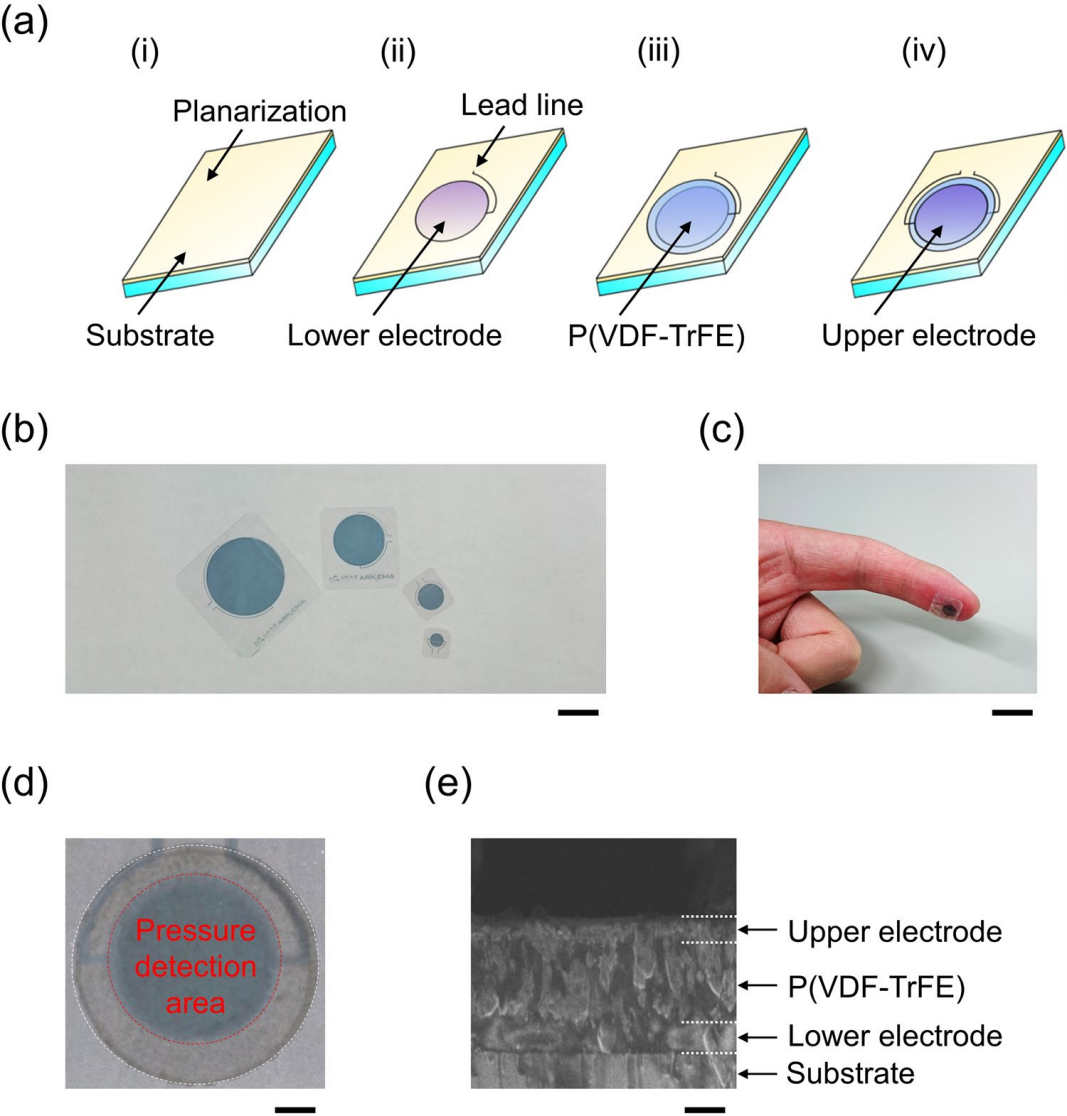
Fabrication of the fully printed vital sensors. (**a**) Schematic of the fully printed vital sensor fabrication process: (i) Formation of the planarization layer on a PEN substrate; (ii) Printing of the lower electrode using PEDOT:PSS; (iii) Patterning of the P(VDF-TrFE) layer; (iv) Printing of the upper electrode using PEDOT:PSS. (**b**) Photographs of the fabricated sensing devices (four different sensor sizes of 4, 20, 75, and 130 mm^2^; scale bar: 10 mm. (**c**) Fabricated vital sensor with high flexibility, laminated on the forefinger. (**d**) Magnified view of the sensor. The white dotted circle indicates the P(VDF-TrFE) layer area and the red dotted circle indicates the pressure detection area; scale bar: 5 mm. (**e**) Cross-sectional SEM image of the printed vital sensor; scale bar: 1 µm.

The fully printed vital sensor for wearable devices studied in this work is based on poly(3,4-ethylenedioxythiophene):poly(4-styrenesulfonate) PEDOT:PSS thin electrodes and a P(VDF-TrFE) layer. Figure 1a shows the fabrication process of the proposed printed vital sensor. This sensor was fabricated entirely by screen printing processes and can be fabricated in various sizes, as shown in Fig. 1b. The minimum size of the pressure detection area of the fabricated sensor was 4 mm^2^. A photograph of the vital sensor device laminated on a forefinger is displayed in Fig. 1c. Figure 1d shows the magnified view of the sensor. The cross-sectional scanning electron microscope (SEM) image of the sensor is shown in Fig. 1e; it shows PEDOT:PSS electrodes and a P(VDF-TrFE) layer with average thicknesses of 500 nm and 2 μm, respectively.

### Homogeneity of the printed piezoelectric polymer layer

**Figure 2:**
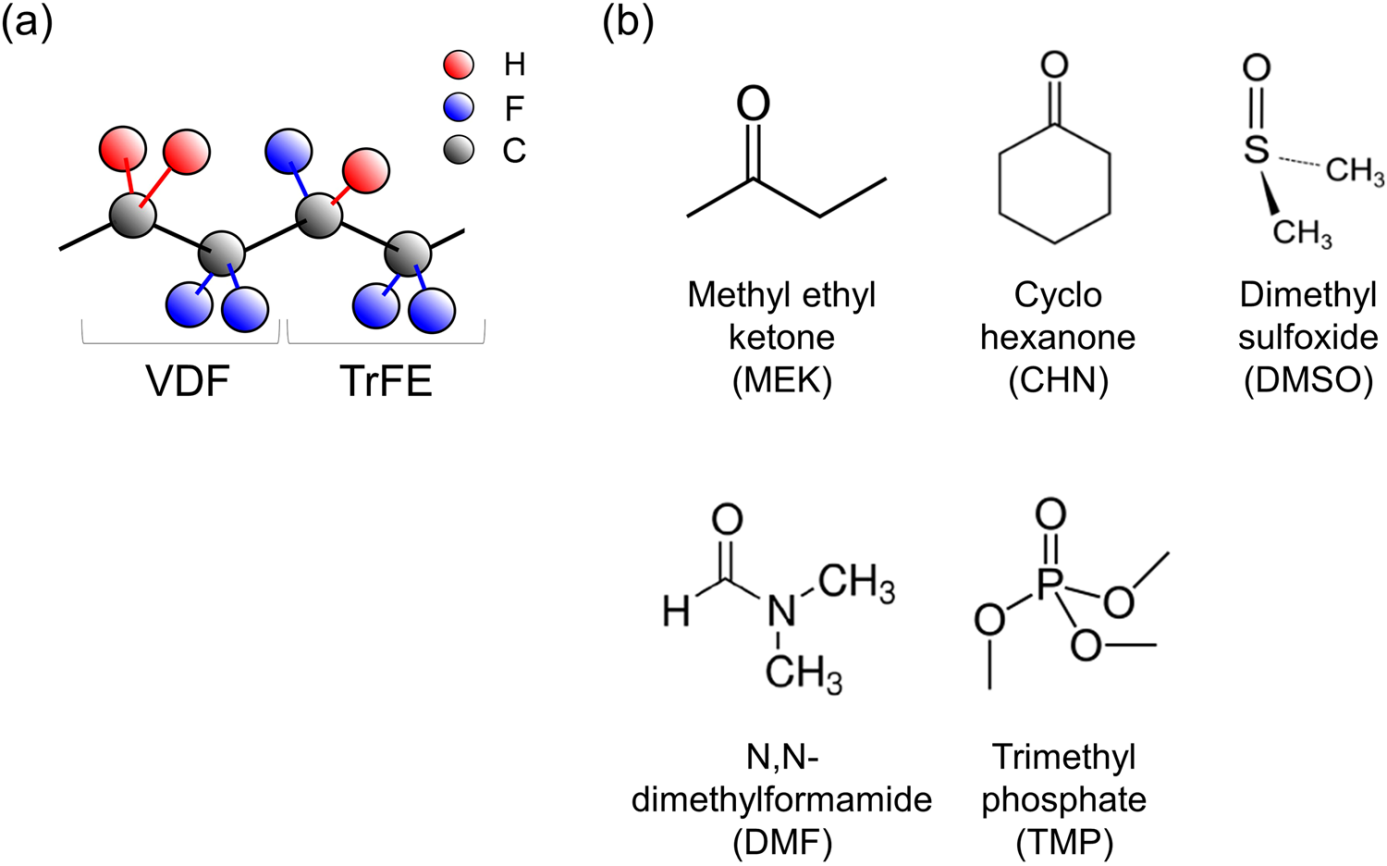
Chemical structures and characteristics of the polar solvent. **(a**) Chemical structure of P(VDF-TrFE) (VDF:TrFE molar ratio of 75:25). (**b**) Chemical structure of the polar solvents used in this study.

**Figure 3:**
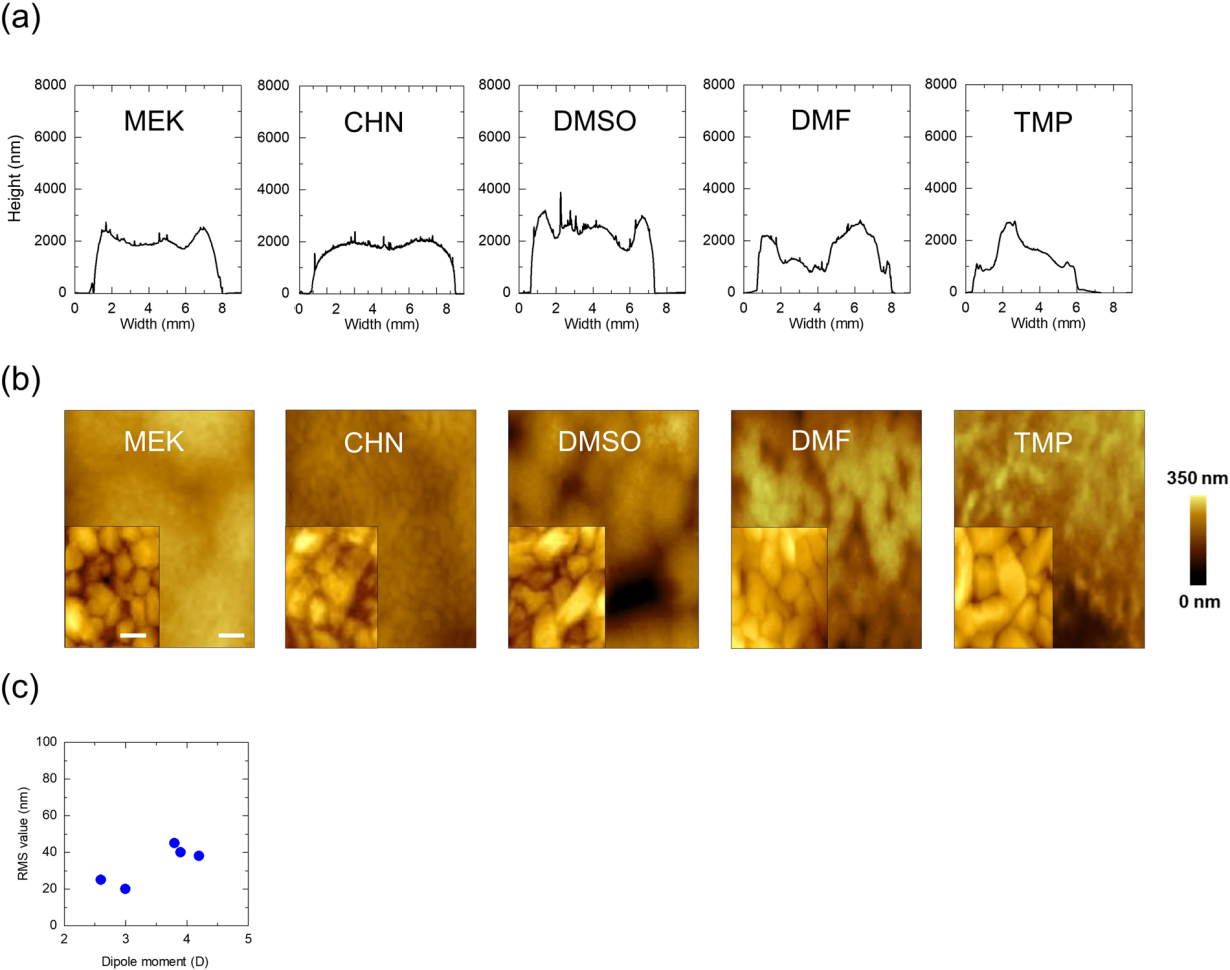
Surface profiles. (**a**) Surface profiles of the printed P(VDF-TrFE) layers using various polar solvents. The thicknesses of the layers were approximately 2 μm. (**b**) Surface AFM images of the P(VDF-TrFE) layers using various polar solvents; scale bar: 1 µm. The inset images are the magnified views; scale bar: 100 nm. (**c**) RMS values estimated from the AFM images as a function of the dipole moments of the polar solvents.

Figure 2a,b show the chemical structures of the P(VDF-TrFE) and the polar solvents, respectively, which are used in the fabrication of the sensor. In Table 1, the dipole moments (Debye; D) calculated using the Gaussian density functional theory (calculation conditions of the quantum chemistry is as follows: B3LYP/6-31 G(d), Approximate methods/Basis functions) are depicted. The dotted lines are only for guidance. Figure 3a shows the surface profiles of the printed P(VDF-TrFE) layers for each solvent; the profiles of the printed layer depend on the polar solvents used. In particular, the P(VDF-TrFE) layer for which Dimethyl sulfoxide (DMSO), N,N-dimethylformamide (DMF), and Trimethyl phosphate (TMP) were used does not tend to form flat layers during screen printing. On the other hand, Methyl ethyl ketone (MEK) and Cyclohexanone (CHN) can form ideally flat ferroelectric layers on printing. The above results demonstrate that a polar solvent with a dipole moment less than approximately 3 D can realise a flat ferroelectric layer when used with P(VDF-TrFE) during screen printing. The detailed printability of the copolymer solution is depicted in Supplementary Fig. S1. This information is crucial for the fabrication of vital sensors. Surface atomic force microscope (AFM) images of the screen-printed P(VDF-TrFE) layer are displayed in Fig. 3b. The dependence of the morphology and crystallinity of the P(VDF-TrFE) layer on the polar solvents could be explicitly observed. These results clearly indicate that the solvents can improve the microscale surface morphology also, as shown in the inset images of Fig. 3b. Moreover, when the dipole moment increased beyond 3 D, the trend of the Root-Mean-Square (RMS) value of the height direction, which was calculated from the AFM images, worsened (see Fig. 3c). Therefore, it was determined that a polar solvent with a relatively low dipole moment is appropriate for the fabrication of highly uniform printed P(VDF-TrFE) layers. In this work, we suggest a polar solvent most suited to P(VDF-TrFE) based on the surface homogeneity and crystallinity for avoiding leakage paths between the printed electrodes and the P(VDF-TrFE) layer.

**Table 1:** Dipole moments and boiling points of the polar solvents used for P(VDF-TrFE).

	MEK	CHN	DMSO	DMF	TMP
Dipole moment (D)	2.6	3.0	3.7	3.8	4.2
Boiling point (°C)	80	155	189	153	197

### Ferroelectricity and piezoelectric responses

**Figure 4:**
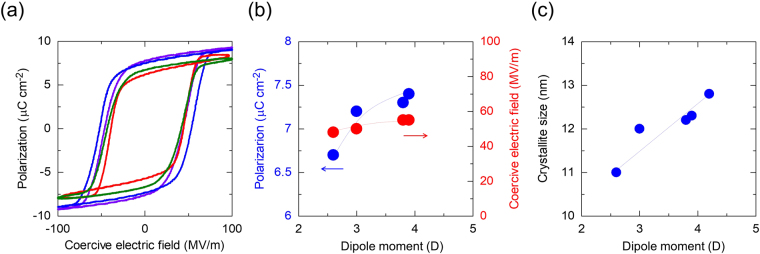
Ferroelectricity and crystallinity. (**a**) P-E hysteresis loops between the polarisation and coercive electric fields for the printed vital sensor with various polar solvents. Red line: MEK, Green line: CHN, Purple line: DMSO, and Blue line: DMF. (**b**) Polarisation and coercive electric fields of the P(VDF-TrFE) layers as a function of the dipole moments of the polar solvents. (**c**) Average crystallite size of the P(VDF-TrFE) layers as a function of the dipole moment.

Figure 4a shows the electric field (*P-E*) loops for the ferroelectric polarisation of the printed vital sensor using various polar solvents. As mentioned above, P(VDF-TrFE) exhibits piezoelectric characteristics owing to its ferroelectricity. Therefore, the P(VDF-TrFE) layer has a piezoelectric voltage response^26–28^. We measured the generated voltages in order to quantify the piezoelectricity. The characteristics of PEDOT:PSS as a printed electrode were similarly calculated and depicted in Supplementary Fig. S2. We had previously confirmed that the resistivity of the PEDOT:PSS layer does not affect the piezoelectricity of the fabricated vital sensor. It should be noted that the *P-E* loop for which TMP was used in the P(VDF-TrFE) layer could not be measured because it did not have fine uniformity (Fig. 3a). The polarization, *P* (µC cm^−2^), and the coercive electric field, *E* (MV m^−1^), are shown in Fig. 4b. In accordance with the changes in the dipole moment, the polarization changed from 6.5 to 7.5 µC cm^−2^ and the coercive field changed from 48 to 53 MV m^−1^. Polarization value of P(VDF-TrFE) typically depends on its crystallinity. The polarization of the printed capacitor fabricated from the PVDF copolymer with MEK as the solvent was slightly lower than that of others due to the dependence on its crystallinity. These results indicate that the polarization of the P(VDF-TrFE) layer in screen printing depends upon the dipole moments of the polar solvents. In Fig. 4c, the crystallite sizes of the P(VDF-TrFE) layer calculated from the X-ray diffraction (XRD) spectra at (110/200) lattice planes are depicted. The average crystallite size, *D*, was estimated as follows:

1 $$D\,(\mathring{\rm A} )=\frac{k{\lambda }_{Cu}}{\beta cos\theta }.$$

Here, *k* and *λ*_*Cu*_ are the Scherrer constant (*k* = 0.95) and the wavelength of the X-ray (*λ* = 1.54 Å, Cu source), respectively. *β* and *θ* are the full width at half maximum (FWHM) values (calculated from the diffraction widths of effective and full crystals) of the actual survey and Bragg angles, respectively. The out-of-plane XRD spectra of the printed P(VDF-TrFE) layer are shown in Supplementary Fig. S3; the crystallite sizes of the P(VDF-TrFE) were in correlation with the dipole moments of the solvents, which is in accordance with the tendency of the results in previous research^40^.

**Figure 5:**
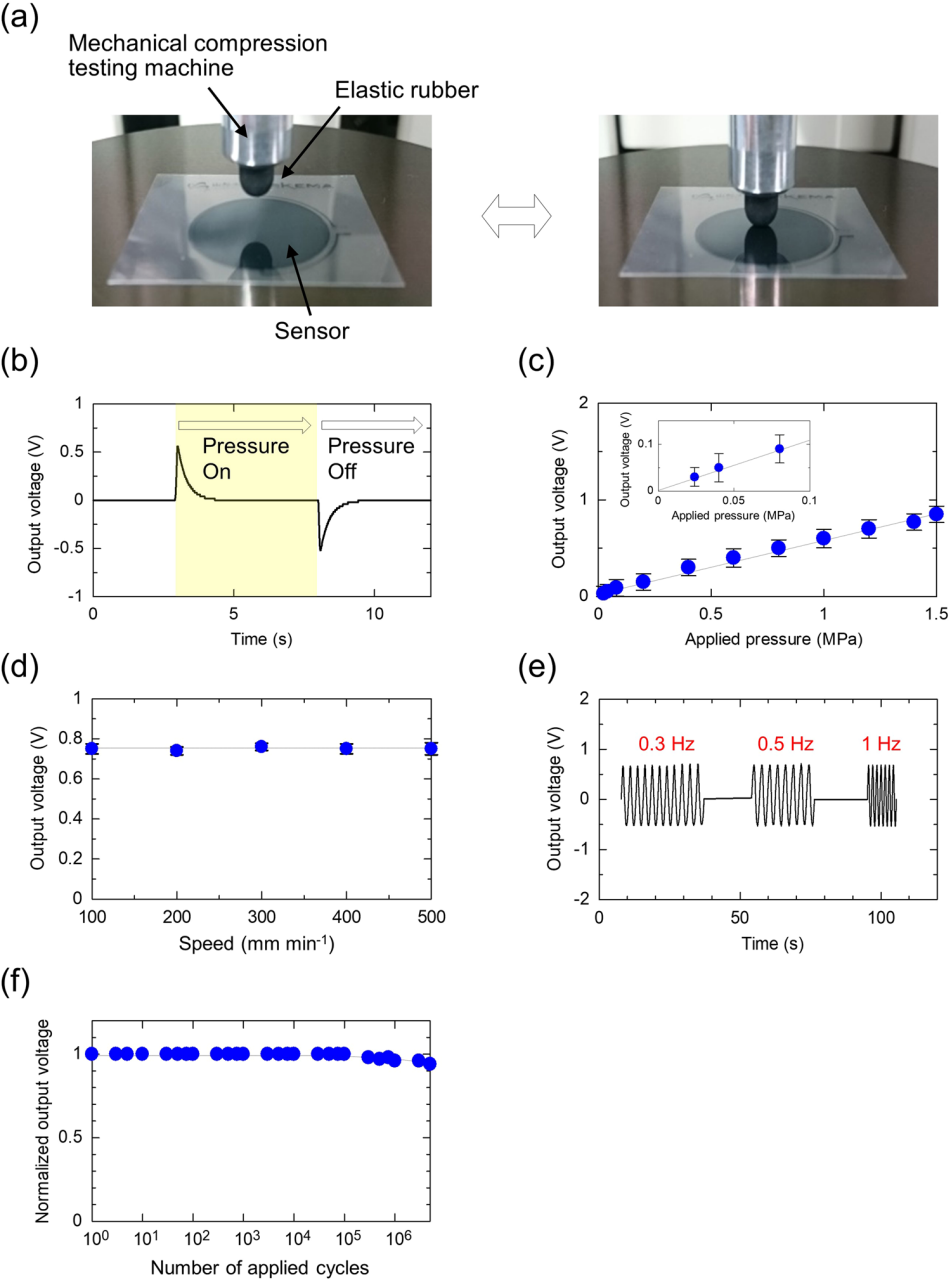
Pressure-sensing of the printed vital sensor. (**a**) Photographs of the pressure-sensing setup for the printed vital sensor using a mechanical compression testing machine. (**b**) Piezoelectric response of the printed sensor. The applied pressure was 0.75 MPa and the area was 1.5 mm^2^. Sample number: 5. (**c**) Plots of the relative changes in output voltage for various applied pressures. The inset graph shows the magnified area of the output voltage from 0 to 0.1 MPa. (**d**) Output voltage of the vital sensor as a function of the pressure at speeds of 100–500 mm min^−1^. The applied pressure was 0.75 MPa. Sample number: 5. (**e**) Frequency response dependence on the applied pressure. The applied pressure was 0.75 MPa. (**f**) Normalised output voltage of the vital sensor as a function of the number of pressure cycles. The applied pressure was 0.75 MPa.

For evaluating the pressure sensitivity of the printed vital sensor, the piezoelectric response was measured by applying 0.025–1.5 MPa pressure. As the CHN solvent could realise the smoothest surface for the P(VDF-TrFE) layer, it was selected for the experiment shown in Fig. 5a displays the photographs of the pressure-sensing setup for the sensor, which uses a mechanical compression testing machine. The piezoelectric response of the sensor is shown in Fig. 5b. Voltage peaks were generated in the positive direction when pressure was applied to the sensor (pressure-on). After the pressure was released, the peaks were generated in the negative direction (pressure-off); the generated voltages on the positive-side corresponded to those on the negative-side. The response time of the sensor (approximately 0.2 s) with the applied pressure is depicted in Supplementary Fig. S4. The plots of the peak positive voltages for various applied pressures are shown in Fig. 5c. The generated voltage indicates a linear dependence on the pressure. This is significant because it demonstrates that the fabricated vital sensor is sensitive to low pressure below 0.1 MPa (inset graph in Fig. 5c); the sensor has sufficient sensitivity for monitoring the human pulse rate and exhibits satisfactory pressure sensitivity^41^. General piezo films such homo- and copolymers generate voltages only as long as the linear regime of the piezoelectric effect does not exist. Our sensor detected the pressure under the piezoelectric effect and monitored the pressure changes. The equation for estimating the piezoelectric constant, *d*_33_, of P(VDF-TrFE) is as follows:

2 $${d}_{33}=\frac{{V}_{gen}{C}_{P(VDF-TrFE)}}{P}.$$

Here, *V*_gen_, *C*_P(VDF − TrFE)_, and *P* are the generated voltage, capacitance of the sensor, and the applied pressure, respectively. In this calculation, *V*_gen_ was estimated using the least-squares method from the slope between the pressure and the generated voltage in Fig. 5c. The measured capacitance of the sensor was 56.4 pF (area of the capacitor (*S)*: 1.5 mm^2^, dielectric constant (*ε)*: 8.5, thickness (*t*): 2000 nm). The calculated constant *d*_33_ was −22.1 pC N^−1^, which approximately corresponds to the theoretical value^22,42–44^. It can be clearly deduced from equation (2) that the generated voltage from the P(VDF-TrFE) layer changed depending upon the variable parameter *P* for the applied pressure. Figure 5d shows voltage responsivity characteristics of the sensor with respect to the rate of application of pressure. The generated voltage does not depend upon the rate of the applied pressure. Our sensor generated an output voltage without dependence on the rate of the applied pressure during the experiments performed in this study. Figure 5e depicts the dependence of the frequency-response characteristics on the pressure. The frequencies of the applied pressure (0.75 MPa) were 0.3, 0.5, and 1 Hz. The response time was approximately 0.2 s, which exceeds the requirement for monitoring of pressure in the blood vessels of the human body^18^. Finally, the results of the mechanical stability are shown in Fig. 5f. The number of pressure cycles was more than 10^6^. Supplementary Fig. S5 shows the additional results of the flexibility and mechanical durability of the vital sensor; a radius of curvature of 1 mm does not affect the ferroelectricity of the sensor. These results establish that the sensor can acquire sustainable and reliable pulse signals due to its high piezoelectric responsivity and mechanical stability.

### Blood pressure wave monitors

**Figure 6:**
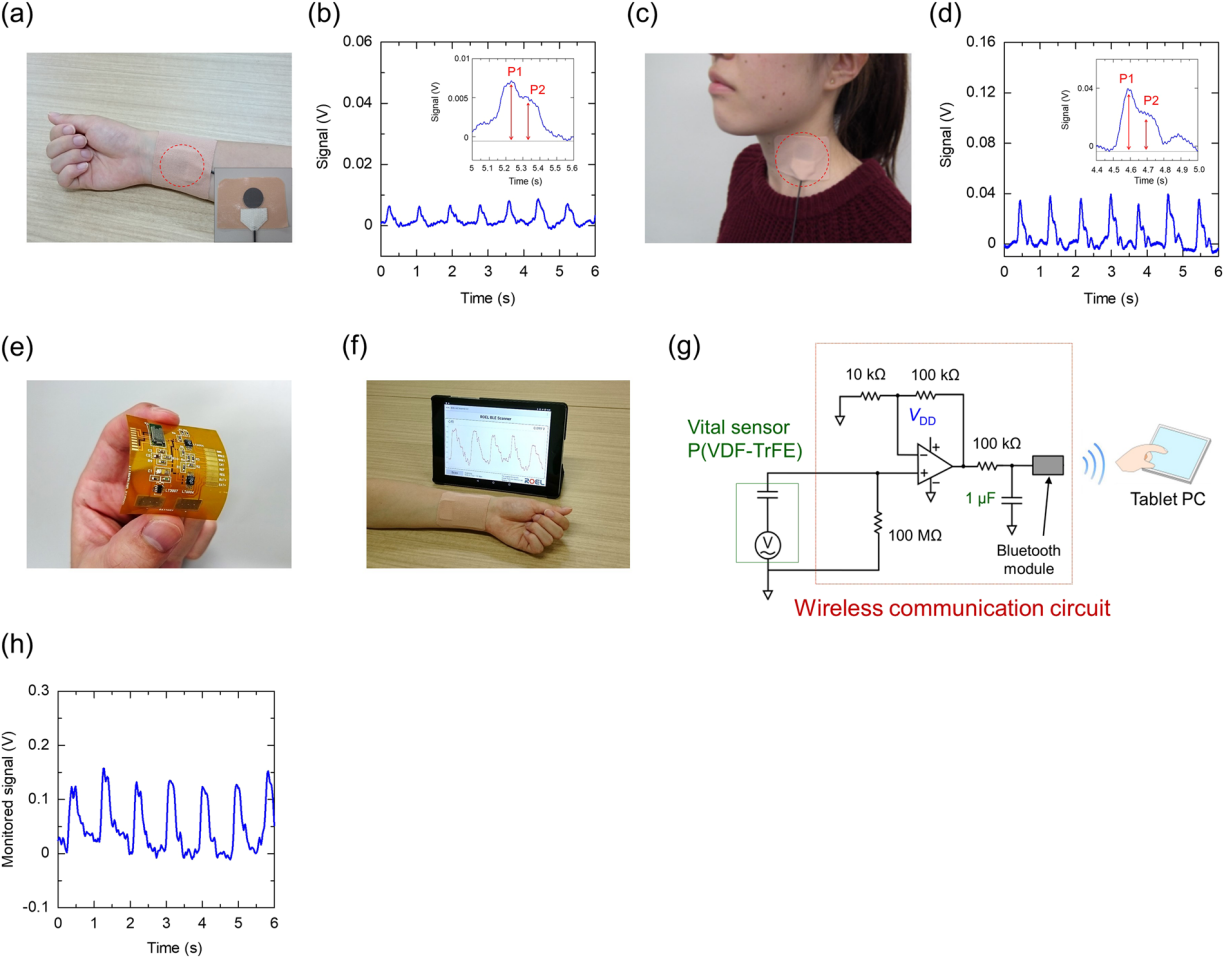
Pulse wave of the radial artery measured using the vital sensor. Photograph of the vital sensor attached to the skin of a volunteer using a skin-compatible adhesive patch on the (**a**) wrist and (**c**) neck. Real-time monitoring of the pulse wave signal from the blood flow: (**b**) at the wrist and (**d**) neck. (**e**) Image of the wireless sensing system setup using the printed vital sensor and wireless communication circuit. (**f**) Photograph of the wireless communication circuit with a tablet PC. (**g**) Wireless communication circuit diagram with a non-inverting amplifier and Bluetooth module. (**h**) Real-time monitoring of the pulse wave signal from the blood flow using the sensor circuit. The sensor circuit was attached to the skin of the wrist by an adhesive patch.

One of the promising applications of the vital sensor is the monitoring of the human pulse wave and its rate for use in healthcare^4,8,45^. Photographs of the fully printed sensor attached to the wrist and neck are displayed in Fig. 6. The demonstrations involved a healthy female volunteer with approval from the institutional review board of the Yamagata University (no. 29-2). In addition, we confirmed that all experiments were performed in accordance with relevant guidelines and regulations of the institutional review board. The sensor was attached to the skin on the wrist and the neck of the volunteer using a skin-compatible adhesive patch, as shown in Fig. 6a,c. We confirmed that there was no allergic reaction, redness, or damage to the skin when the sensor was attached. The changes in the signal owing to the variations in the pressure associated with the pulse wave from the blood flow are depicted in Fig. 6b,d; from the magnified views provided in the insets of the figure, it can be seen that the changes in the amplitude of the signal with the blood flow can be clearly measured. The pulse rate of the volunteer was determined to be 70 min^−1^ using the sensor. The human pulse wave spectra are divided into P1, a novice wave, and P2, a reflected wave. P1 occurs during cardiac shrinkage and P2 develops when the novice wave is reflected from a peripheral blood artery^46,47^. The above-mentioned results, particularly in Fig. 6b,d, establish that the sensor can monitor P1 and P2. By comparing the amplitudes of P1 and P2, the radial artery augmentation index (AI) can be calculated as follows:

3 $${\rm{AI}}\,( \% )=\frac{P2}{P1}\times 100.$$

The AI value is effective in determining the hardness of human blood vessels^48,49^. In this study, the experimental AI value in Fig. 6d was 62.5%, which generally indicates a healthy female. This established that the sensor could acquire information regarding arteriosclerosis and heart diseases effectively. These findings can be utilised for realising the development of healthcare applications using printed sensor devices attached to the skin. Figure 6e displays the designed wireless sensing system consisting of the printed vital sensor and wireless communication circuit for the wireless measurement of the pulse rate. Figure 6f displays a photograph of the wireless communication circuit with a tablet PC. The wireless sensing system, including the vital sensor and the wireless communication circuit, can send pulse wave/rate data to an electric display device such as a tablet PC, as shown in Fig. 6f. Figure 6g presents the circuit diagram of the wireless communication circuit, which uses a non-inverting amplifier along with an operational amplifier (op-amp) and high/low-pass filters. The monitored vital signal is sent to a tablet PC through a wireless Bluetooth Low Energy (BLE) module. In the wireless communication circuit, the amplification factor was set to 10 by using 10 and 100 kΩ resistors along with an op-amp. The sampling frequency of this sensor circuit was set to 10 ms. The schematic of the wireless circuit system with the printed sensor and a seat-type cell battery before attachment to the skin is displayed in Supplementary Fig. S6. In Fig. 6h, the monitoring of the pulse wave/rate by the wireless sensor circuit is demonstrated. Despite using wireless communication, data was clearly displayed on the tablet PC. The amplification factor of the monitored signal strength was approximately 10 by comparing with Fig. 6b. The pulse rate of the volunteer was estimated to be 65 min^−1^ using the sensor circuit. These results establish that the vital sensor and the developed circuit increase potentials of next-generation technologies, such as wearable electronics, for healthcare applications.

## Discussion

The screen-printed ferroelectric polymer layer in the vital sensor was very smooth owing to the optimisation of the polar solvents. This surface morphology and profile are significant in forming each layer of the printed sensor. TMP could not realise a smooth layer; hence, leakage paths between the printed electrodes and P(VDF-TrFE) layer could not be avoided. The differences in the homogeneity of the printed P(VDF-TrFE) layer were attributed to the dipole moments. The solvents with lower dipole moments could not provide the thermal energy required for the configurational and conformational entropically favored states. Rapid solvent evaporation can form a flat surface because the entropy is not transferred to the P(VDF-TrFE) layer from the solvents. On the other hand, solvents with high dipole moments form rougher surfaces because slow solvent evaporation transfers thermal energy to the P(VDF-TrFE)^32,50,51^. M. Benz *et al*. discussed the effect of polar solvents on the roughness of films prepared from PVF_2_ polymers and predicted that solvents with higher dipole densities might form rougher surfaces^52^. The improved P(VDF-TrFE) layer achieved satisfactory ferroelectricity, which was approximately 7.0 μC cm^−2^ of polarisation, and demonstrated obvious piezoelectric responses against an applied low pressure of 0.025 MPa. The sensor exhibited sufficient sensitivity for monitoring the pulse rate and had superior pressure sensitivity. The piezoelectricity of P(VDF-TrFE) depended on its crystallinity. In the fabrication of the P(VDF-TrFE) layer, we reduced the temperature of samples by the more common slow cooling process after annealing at 135 °C for 1 h. Fine piezoelectricity and homogeneity cannot be realised if it is cooled down rapidly^53–55^.

The sensor was attached to a human wrist and neck to demonstrate the sensing and measurement of the pulse rate by real-time scanning. Additionally, the printed sensor was connected to a flexible substrate with a communication amplifier circuit for the wireless detection of the pulse wave/rate. The sensor circuit satisfactorily detected the pulse rate and displayed the data clearly on a tablet PC.

In conclusion, we fabricated a fully printed vital sensor made of P(VDF-TrFE), which can be applied for pulse wave/rate monitoring on the skin. The printed sensor exhibited high responsivity to the applied pressure and mechanical stability. Moreover, we succeeded in wireless monitoring of the pulse wave using the sensor. The information obtained from the fabrication of the fully printed vital sensor used in this study further illustrates the potential of these devices in fabricating novel electronic devices such as wearable applications.

## Methods

### Fabrication process of the fully printed vital sensor

The fully printed vital sensor was fabricated on a 50-μm thick (Q65HA, DuPont) poly(ethylene naphthalate) (PEN) film substrate and fixed to a glass carrier. A cross-linkable poly(4-vinyl-phenol) (PVP) (436224, Sigma-Aldrich) solution consisting of a mixture of PVP and melamine resin (418560, Sigma-Aldrich) with 1-methoxy-2-propyl acetate (Kanto Chemicals 01948-00) as the solvent was spin-coated onto the PEN film as the planarization layer. The lower electrode made of PEDOT:PSS (Clevios SV4 STAB, Heraeus) was formed on the planarization layer by screen printing (MT320T, Micro-tec, Japan), and annealed at 150 °C for 30 min; the thickness was approximately 500 nm. Further, a 2000-nm layer of poly(vinylidene fluoride-co-trifluoroethylene) [P(VDF-TrFE)] (62-010, Piezotech, France; VDF:TrFE molar ratio of 75:25) was formed by screen printing and annealed at 135 °C for 1 h^21^. P(VDF-TrFE) was dissolved in several polar solvents at concentrations of 10 wt%. The upper electrode of the sensor was formed with PEDOT:PSS using a screen printer and annealed at 135 °C for 30 min. Finally, a 200-nm layer of Cytop (CTX-809A, Asahi Glass, Japan) was spin-coated as the passivation layer and annealed at 100 °C for 10 min.

### Screen printing Process

The electrodes and the P(VDF-TrFE) layer were formed by screen printing. The moving speed of the squeeze was 50 mm min^−1^. Room temperature was maintained for stable and reliable printing. The clearance between the substrate and mask was 1.2 mm.

### Performance measurements

All electric characteristics were measured under atmospheric conditions using an oscilloscope (MDO3000, Tektronix) and a waveform generator (AFG3101C, Tektronix). The *P*-*E* loops were estimated by the Sawyer–Tower method. The interface and surface morphology of the P(VDF-TrFE) layer were observed by SEM (7600-FE, JEOL, Japan) and AFM (5500, Agilent). The piezoelectricity of the sensor was measured by a mechanical compression testing machine (STD-203NB, Imada Seisakusho, Japan). We conducted XRD (SmartLab, Rigaku, Japan) measurements on the P(VDF-TrFE) layer to assess the crystallinity and analyse the crystal structures.

### Fabrication of the wireless communication circuit

The Cu electrode of the wireless communication circuit was fabricated on a flexible polyimide film by photolithography. For the amplifier circuit, an operational amplifier (LT6004, Linear Technology), chip resistors (2 mm × 1.2 mm), and a chip capacitor (2 mm × 1.2 mm) were mounted on the polyimide substrate by soldering. Further, the printed vital sensor and a sheet-type cell battery of 25 mAh (FDK Corporation, Japan) were connected to the substrate of the circuit using conductive tape (ALCARE Co., Ltd., Japan). A wireless BLE module (EYSGJNZWY, Taiyo Yuden Co., Ltd.) was similarly mounted on the substrate for transmitting the measured pulse rate data to a PC tablet. This sensor circuit was attached to the skin near the wrist of a volunteer using a skin-compatible adhesive patch. For the measurement of human pulse rate, the ethics statement in this manuscript includes a sentence confirming that written informed consent was obtained from all subjects.

### Data availability

All data generated or analysed during this study are included in this published article (and its Supplementary Information files). In this manuscript, demonstrations involved a volunteer with approval from the institutional review board of the Yamagata University (no. 29-2). The specific consents from the authors and volunteer have been obtained to publish the information in an open-access online publication. The authors confirmed that all experiments were performed in accordance with relevant guidelines and regulations of the institutional review board.

## Electronic supplementary material

Supplementary Information

## References

[1] Gao W (2016). Fully integrated wearable sensor arrays for multiplexed *in situ* perspiration analysis. Nature.

[2] Lorwongtragool P, Sowade E, Watthanawisuth N, Baumann RR, Kerdcharoen T (2014). A Novel Wearable electronic nose for healthcare based on flexible printed chemical sensor array. Sensors.

[3] Yokota T (2016). Ultraflexible organic photonic skin. Sci. Adv..

[4] Pang C (2012). A flexible and highly sensitive strain-gauge sensor using reversible interlocking of nanofibers. Nat. Mater..

[5] Soh PJ, Vandenbosch GAE, Mercuri M, Schreurs DMM-P (2015). Wearable wireless health monitoring: Current developments, challenges, and future trends. IEEE Microw. Mag..

[6] Chen LY (2014). Continuous wireless pressure monitoring and mapping with ultra-small passive sensors for health monitoring and critical care. Nat Commun..

[7] Dagdeviren C (2014). Conformable amplified lead zirconate titanate sensors with enhanced piezoelectric response for cutaneous pressure monitoring. Nat. Commun..

[8] Zirkl M (2011). An all-printed ferroelectric active matrix sensor network based on only five functional materials forming a touchless control interface. Adv. Mater..

[9] Tien NT, Trung TQ, Seoul YG, Kim DI, Lee N-E (2011). Physically responsive field-effect transistors with giant electromechanical coupling induced by nanocomposite gate dielectrics. ACS NANO.

[10] Yan C (2014). Highly stretchable piezoresistive graphene–nanocellulose nanopaper for strain sensors. Adv. Mater..

[11] Wang Y (2014). Wearable and highly sensitive graphene strain sensors for human motion monitoring. Adv. Mater..

[12] Imani S (2016). A wearable chemical–electrophysiological hybrid biosensing system for real-time health and fitness monitoring. Nat. Commun..

[13] Yamada T (2011). A stretchable carbon nanotube strain sensor for human-motion detection. Nat. Nanothenol..

[14] Fukuda K (2014). Fully-printed high-performance organic thin-film transistors and circuitry on one-micron-thick polymer films. Nat. Commun..

[15] Minari T (2014). Room-temperature printing of organic thin-film transistors with π-Junction gold nanoparticles. Adv. Funct. Mater..

[16] Sharma T, Je S-S, Gill B, Zhanga JXJ (2012). Patterning piezoelectric thin film PVDF-TrFE based pressure sensor for catheter application. Sensor. Actuat. A-Phys..

[17] Adhikary P, Garain S, Mandal D (2015). The co-operative performance of a hydrated salt assisted sponge like P(VDF-HFP) piezoelectric generator: an effective piezoelectric based energy harvester. Phys. Chem. Chem. Phys..

[18] Park SH, Lee HB, Yeon SM, Park J, Lee NK (2016). Flexible and stretchable piezoelectric sensor with thickness-tunable configuration of electrospun nanofiber mat and elastomeric substrates. ACS Appl. Mater. Interfaces.

[19] Persano L (2013). High performance piezoelectric devices based on aligned arrays of nanofibers of poly(vinylidenefluoride-co-trifluoroethylene). Nat. Commun..

[20] Wang YR, Zheng JM, Ren GY, Zhang PH, Xu C (2011). A flexible piezoelectric force sensor based on PVDF fabrics. Smart Mater. Struct..

[21] Sekine T (2016). Fully printed and flexible ferroelectric capacitors based on a ferroelectric polymer for pressure detection. Jpn. J. Appl. Phys..

[22] Lovinger AJ (1983). Ferroelectric polymer. Science.

[23] Dietze M, Es-Souni M (2008). Structural and functional properties of screen-printed PZT–PVDF-TrFE composites. Sensor. Actuat. A-Phys..

[24] Zhang QM (2002). An all-organic composite actuator material with a high dielectric constant. Nature.

[25] Bae S-H (2013). Graphene-P(VDF-TrFE) multilayer film for flexible applications. ACS NANO.

[26] Meng N, Zhu X, Mao R, Reece MJ, Bilottia E (2017). Nanoscale Interfacial Electroactivity in PVDF/PVDF-TrFE Blended Films with Enhanced Dielectric and Ferroelectric Properties. J. Mater. Chem. C.

[27] Bhavanasi. V, Kusuma DY, Lee PS (2014). Polarization Orientation, Piezoelectricity, and Energy Harvesting Performance of Ferroelectric PVDF-TrFE Nanotubes Synthesized by Nanoconfi nement. Adv. Energy Mater..

[28] Kim RH (2014). Non-volatile organic memory with sub-millimetre bending radius. Nat. Commun..

[29] Zhang X, Xu H, Zhang Y (2011). Temperature dependence of coercive field and fatigue in poly(vinylidenefluoride-trifluoroethylene) copolymer ultra-thin films. J. Phys. D: Appl. Phys..

[30] Xu H, Cheng Z-Y, Olson D, Mai T, Zhang QM (2001). Ferroelectric and electromechanical properties of poly (vinylidene-fluoride-trifluoroethylene-chlorotrifluoroethylene) terpolymer. Appl. Phys. Lett..

[31] Simoes R, Rodriguez-Perez M, de Saja J, Constantino C (2009). Thermomechanical characterization of PVDF and P(VDF-TrFE) blends containing corn starch and natural rubber. J. Therm. Anal. Calorim..

[32] Sussner H, Dransfeld K (1979). Der piezoelektrische Effekt in Polyvinyliden Fluorid und seine Anwendungen. Colloid Polym. Sci..

[33] Marcus MA (1982). Ferroelectric polymers and their applications. Ferroelectrics.

[34] DeRossi D, Dario P (1983). Biomedical applications of piezoelectric and pyroelectric polymers. Ferroelectrics.

[35] DeReggi AS (1984). Transduction phenomena in ferroelectric polymers and their role in biomedical applications. Ferroelectrics.

[36] Nitsche W, Thunker R (1987). Application of the piezo-electric effect in measuring the arterial pressure pulse. Ferroelectrics.

[37] Siivola J (1989). New noninvasive piezoelectric transducer for recording of respiration, heart rate and body movements. Med. Biol. Eng. Comput..

[38] Chen Y, Wang L, Ko WH (1990). A piezopolymer finger pulse and breathing wave sensor. Sensor. Actuat. A-Phys..

[39] Knotts G, Bhaumik A, Ghosh K, Guha S (2014). Enhanced performance of ferroelectric-based all organic capacitors and transistors through choice of solvent. Appl. Phy. Lett..

[40] Kim J (2017). High-Performance Piezoelectric, Pyroelectric, and Triboelectric Nanogenerators Based on P(VDF-TrFE) with Controlled Crystallinity and Dipole Alignment. Adv. Funct. Mater..

[41] Zang Y (2015). Flexible suspended gate organic thin-film transistors for ultra-sensitive pressure detection. Nat. Commun..

[42] Oliveira F (2014). Process Influences on theStructure, Piezoelectric, and Gas-Barrier Properties of PVDF-TrFE Copolymer. J. Polym. Sci. B Polym. Phys..

[43] Omote K, Ohigashi H, Koga K (1997). Temperature dependence of elastic, dielectric, and piezoelectric properties of “single crystalline” films of vinylidene fluoride trifluoroethylene copolymer. J. Appl. Phys..

[44] Bune AV (1999). Piezoelectric and pyroelectric properties of ferroelectric Langmuir–Blodgett polymer films. J. Appl. Phys..

[45] Schwartz G (2013). Flexible polymer transistors with high pressure sensitivity for application in electronic skin and health monitoring. Nat. Commun..

[46] Park DY (2017). Self-powered real-time arterial pulse monitoring using ultrathin epidermal piezoelectric sensors. Adv. Mater..

[47] Nichols WW (2005). Clinical measurement of arterial stiffness obtained from noninvasive pressure waveforms. Am. J. Hypertens..

[48] Yamashina A, Kobayashi H, Takazawa K, Shindo N, Tanaka N (2007). Relationship between radial and central arterial pulse wave and evaluation of central aortic pressure using the radial arterial pulse wave. Hypertens. Res..

[49] Lee H-Y, Oh B-H (2010). Aging and Arterial Stiffness. Circ. J..

[50] Yoo M, Frank CW, Mori S, Yamaguchi S (2004). Interaction of Poly(vinylidene fluoride) with Graphite Particles. 2. Effect of Solvent Evaporation Kinetics and Chemical Properties of PVDF on the Surface Morphology of a Composite Film and Its Relation to Electrochemical Performance. Chem. Mater..

[51] Shi Y, Liu J, Yang Y (2000). Device performance and polymer morphology in polymer light emitting diodes: The control of thin film morphology and device quantum efficiency. J. Appl. Phys..

[52] Benz M, Euler WB, Gregory OJ (2001). The Influence of Preparation Conditions on the Surface Morphology of Poly(vinylidene fluoride) Films. Langmuir.

[53] Singh D, Garg A (2014). Cooling rate controlled microstructure evolution and reduced coercivity in P(VDF-TrFE) devices for memory applications. Org. Electron..

[54] Ren G, Cai F, Li B, Zheng J, Xu C (2013). Flexible Pressure Sensor Based on a Poly(VDF-TrFE) Nanofiber Web. Macromol. Mater. Eng..

[55] Adamowski JC, Buiochi F, Higuti RT (2010). Ultrasonic material characterization using large-aperture PVDF receivers. Ultrasonics.

