# Author Correction: Fully Printed Wearable Vital Sensor for Human Pulse Rate Monitoring using Ferroelectric Polymer

**DOI:** 10.1038/s41598-018-24809-x

**Published:** 2018-04-18

**Authors:** Tomohito Sekine, Ryo Sugano, Tomoya Tashiro, Jun Sato, Yasunori Takeda, Hiroyuki Matsui, Daisuke Kumaki, Fabrice Domingues Dos Santos, Atsushi Miyabo, Shizuo Tokito

**Affiliations:** 1 Research Center for Organic Electronics (ROEL), Graduate School of Science and Engineering, Yamagata University,Yonezawa, Yamagata, 992-8510 Japan; 2 Arkema-Piezotech, 63493 Pierre-Benite Cedex, France; 3 Arkema K. K., 93, Chudoji, Awatacho, Shimogyo, Kyoto, 600-8815 Japan

Correction to: *Scientific Reports* 10.1038/s41598-018-22746-3, published online 13 March 2018

The version of this Article previously published contained lower resolution images for Figures 1, 2, 3, 4, 5 and 6. This has now been corrected in the PDF and HTML versions of the paper.

