# Supplementary material for: Fully Printed Wearable Vital Sensor for Human Pulse Rate Monitoring using Ferroelectric Polymer
Source: Sci Rep. 2018 Mar 13;8:4442. doi: 10.1038/s41598-018-22746-3 (PMC5849614; doi:10.1038/s41598-018-22746-3)
Supplement: Supplementary file 1 — Supplementary Information [file 41598_2018_22746_MOESM1_ESM.docx]

Supplementary Information

**Fully Printed Wearable Vital Sensor for Human Pulse Rate Monitoring using Ferroelectric Polymer**

Tomohito Sekine^1*^, Ryo Sugano^1^, Tomoya Tashiro^1^, Jun Sato^1^, Yasunori Takeda^1^, Hiroyuki Matsui^1^, Daisuke Kumaki^1^, Fabrice Domingues Dos Santos^2^, Atsushi Miyabo^3^, & Shizuo Tokito^1*^

^1^Research Center for Organic Electronics (ROEL), Graduate School of Science and Engineering, Yamagata University, Yonezawa, Yamagata 992-8510, Japan

^2^Piezotech S. A. S., Arkema-CRRA, 63493 Pierre-Benite Cedex, France

^3^Arkema K. K., Kyoto 600-8815, Japan

* Corresponding author: E-mail: tomohito@yz.yamagata-u.ac.jp

Supplementary Figure S1 | Cross-sectional images of the printed P(VDF-TrFE) layer with screen printing method.

Supplementary Figure S2 | Surface roughness of the conductive polymer layer, PEDOT:PSS.

Supplementary Figure S3 | Estimation crystallinity of the P(VDF-TrFE) layers with XRD.

Supplementary Figure S4 | Piezo response time of the fabricated vital sensor.

Supplementary Figure S5 | Flexibility and mechanical durability of the vital sensor.

Supplementary Figure S6 | Photograph of the wireless circuit system with the printed sensor and seat-type cell.


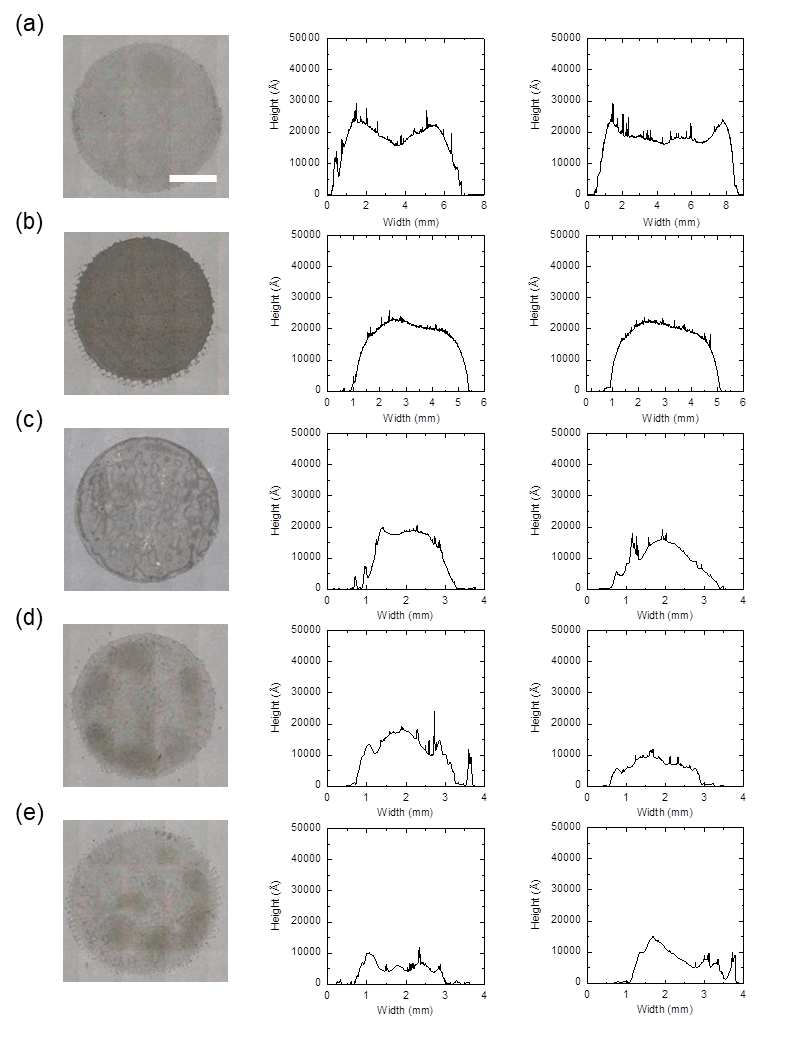


**Supplementary Figure S1 | Surface profile images of the printed P(VDF-TrFE) layer with screen printing method.**

The printed P(VDF-TrFE) layer of (a): MEK, (b): CHN, (c): DMSO, (d): DMF, and (e): TMP. The thickness of the layer was approximately 2 μm. The surface profile images were estimated by scanning on several areas of the layers. The layers were annealed at 135 °C per 1 h. Scare bar, 5 mm.


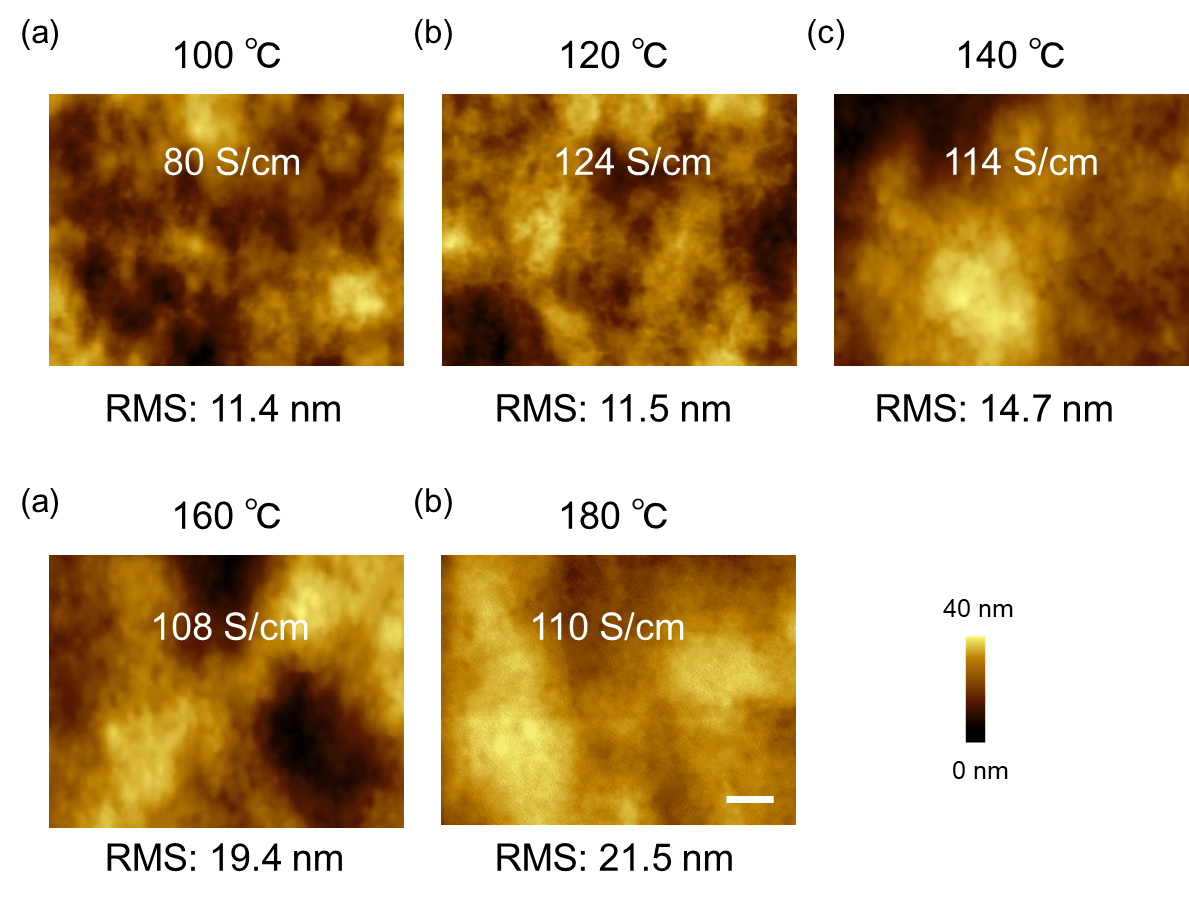


**Supplementary Figure S2 | Surface roughness of the conductive polymer layer, PEDOT:PSS.**

Atomic force microscopy (AFM) was used to measure the root-mean-square (RMS) surface roughness of the individual layers in the PEDOT:PSS which annealed at (a) 100 °C, (b) 120 °C, (c) 140 °C, (d) 160 °C, and (e) 180 °C. The inset values are conductivity of the several layers after annealing.


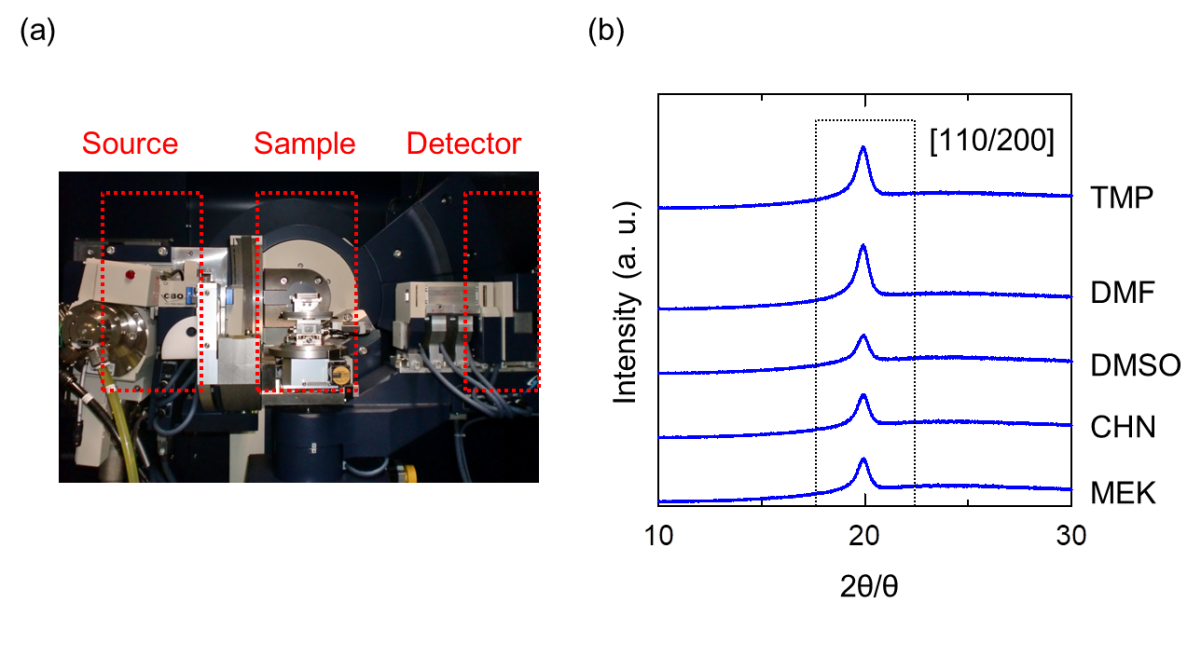


**Supplementary Figure S3 | Estimation crystallinity of the P(VDF-TrFE) layers with XRD.**

(a) Photograph of the XRD measurement for the printed P(VDF-TrFE) layers. (b) Measured XRD patterns of the several P(VDF-TrFE) layers.


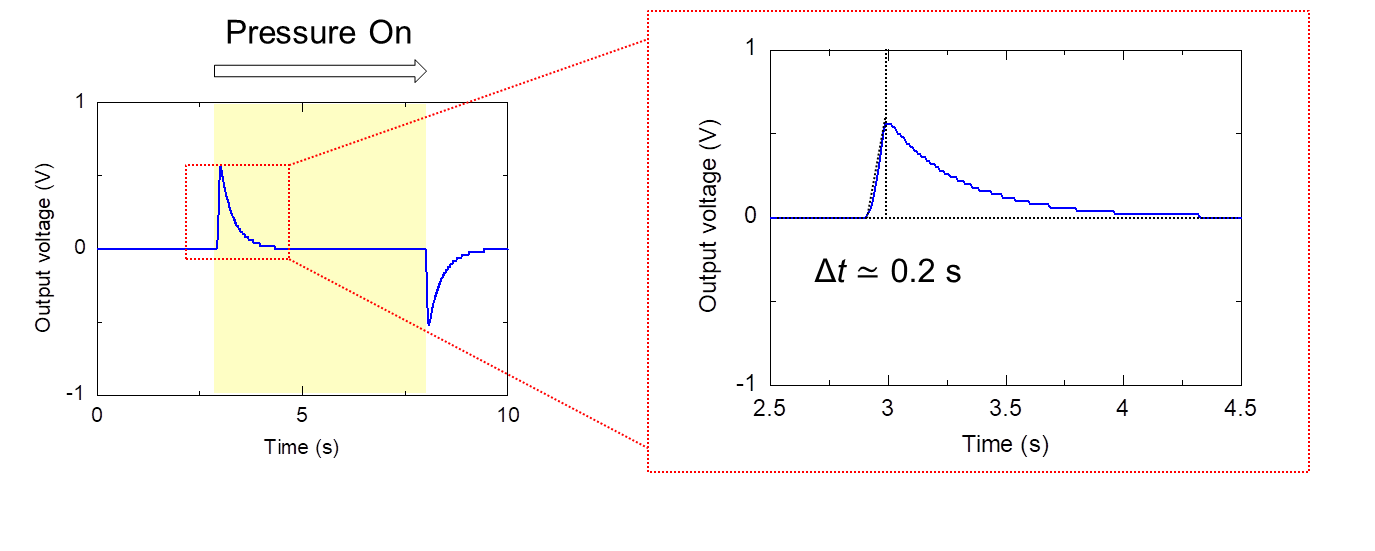


**Supplementary Figure S4 | Piezo response time of the fabricated vital sensor.**

Voltage peaks were generated in the positive direction, when pressure was applied to the sensor. After the pressure was released, the peaks were generated in the negative direction (Left-side). The magnified image of response time of the sensor against applied pressure is shown in right-side. Applied pressure was 0.75 MPa.


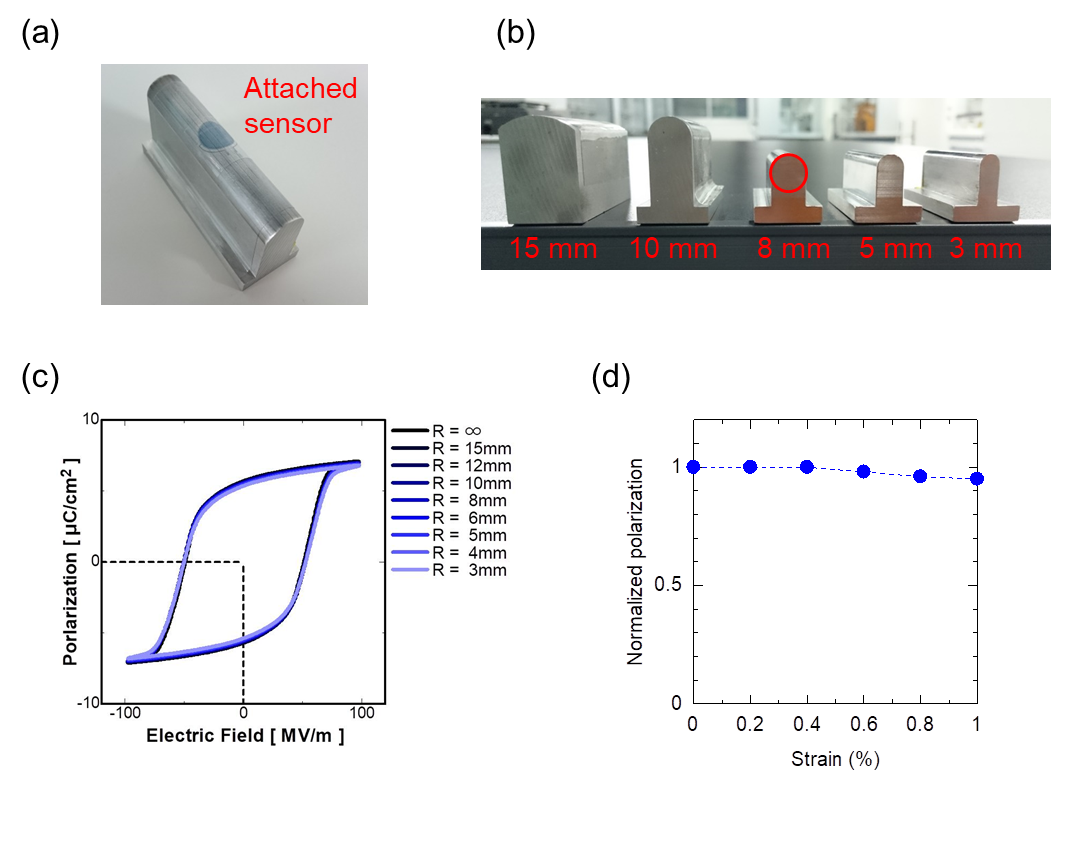


**Supplementary Figure S5 | Flexibility and mechanical durability of the vital sensor.**

(a) Schematic image of the attached vital sensor for a metal stage. (b) Photograph of the several metal stages. The radius of curvature was 15, 10, 8, 5, and 3 mm, respectively. (c) The P-E curves of the fabricated vital sensors under strain. (d) Normalized polarization of the vital sensor as a function of stain. Values of strain were estimated from several radius of curvature and the thickness of the film substrate of the sensor.


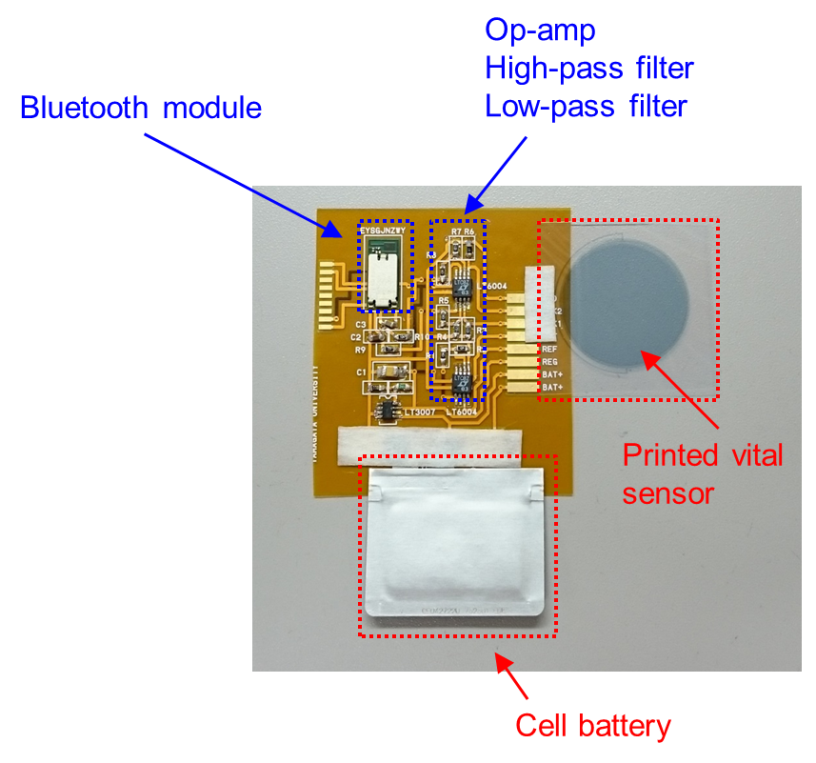


**Supplementary Figure S6 | Photograph of the wireless circuit system with the printed sensor and seat-type cell.**

The printed sensor and a seat-type cell were mounted on the film substrate by an adhesive tape. Capacity of the sheet-type cell battery was 25 mAh. This wireless system was attached to the skin near the wrist of a volunteer using a skin-compatible adhesive patch.
